# Low threshold Rhodamine-doped whispering gallery mode microlasers fabricated by direct laser writing

**DOI:** 10.1038/s41598-017-09293-z

**Published:** 2017-08-17

**Authors:** Nathália B. Tomazio, Leonardo De Boni, Cleber R. Mendonca

**Affiliations:** São Carlos Institute of Physics, University of São Paulo, PO Box 369, 13560-970 São Carlos, SP Brazil

## Abstract

The combination of the outstanding properties of whispering gallery modes with both the flexibility and ease of processing of polymers is particularly attractive for photonics applications. However, the versatile fabrication of polymeric nano/microdevices with the desired photonic performance has proven challenging. Here, we report on lasing in Rhodamine B doped whispering gallery mode microcavities fabricated by direct laser writing via two-photon polymerization. Threshold pump energies as low as 12 nJ were achieved for free-space pulsed excitation at 532 nm. To the best of our knowledge, this is the lowest laser threshold attained for microcavities fabricated in a single step of femtosecond laser writing, a remarkable feat that stands out from other fabrication methods.

## Introduction

Pioneering studies back in the sixties demonstrated the potential of many dyes as active medium for the development of tunable coherent light sources, covering the spectrum from ultraviolet to infrared wavelengths^1–3^. From these early findings forward, many different setups were investigated. Flashlight and high intensity lasers pumping were tested, with threshold excitation of tens of kW/cm^2^ ^4, 5^. The high threshold excitation intensities reported for dye laser setups from the past century were mostly caused by misalignment and successive absorptive/scattering losses inside the cavity, which have been overcome with the development of mirrorless optical devices at micro/nanoscale. Among the emerging microdevices, the so-called whispering gallery mode (WGM) microcavities stand out. Their unique characteristics, such as narrow spectral linewidth, extremely small mode volume and very high power density make them promising tools for the realization of low threshold lasers^6^. In particular, microcavities made of polymers come up with additional advantages, e.g. ease of processing and shaping, low cost and their huge variety of properties^7^.

Much effort has been directed towards the fulfillment of high performance organic microdevices, with the material and fabrication technique to be chosen according to the intended application^8–14^. Femtosecond laser induced two-photon polymerization (2PP) has been proven to be a powerful tool for high-precision 3D microfabrication of organic materials^15–17^. Such technique benefits from the nonlinear nature of the two photon absorption process, which translates into spatial confinement of the photo-driven polymerization^18^. Besides, organic dyes as well as other active material can be directly incorporated into the polymeric matrix previously to fabrication, thus reinforcing the versatility of the technique^19–21^. Here, lasing activity from Rhodamine B (RhB) doped whispering gallery mode microcavities fabricated via two-photon polymerization is reported. The host material is an acrylic based polymeric hollow microcylinder exhibiting good structural quality and smooth sidewall surfaces (Q-factor of 1 × 10^5^ for undoped microcavities around 1550 nm)^22^. The giant power density achieved within the microcavities, combined with the high fluorescence quantum yield of RhB, gives rise to a lasing threshold as low as 12 nJ for free space pulsed excitation at 532 nm.

## Results and Discussions

**Figure 1:**
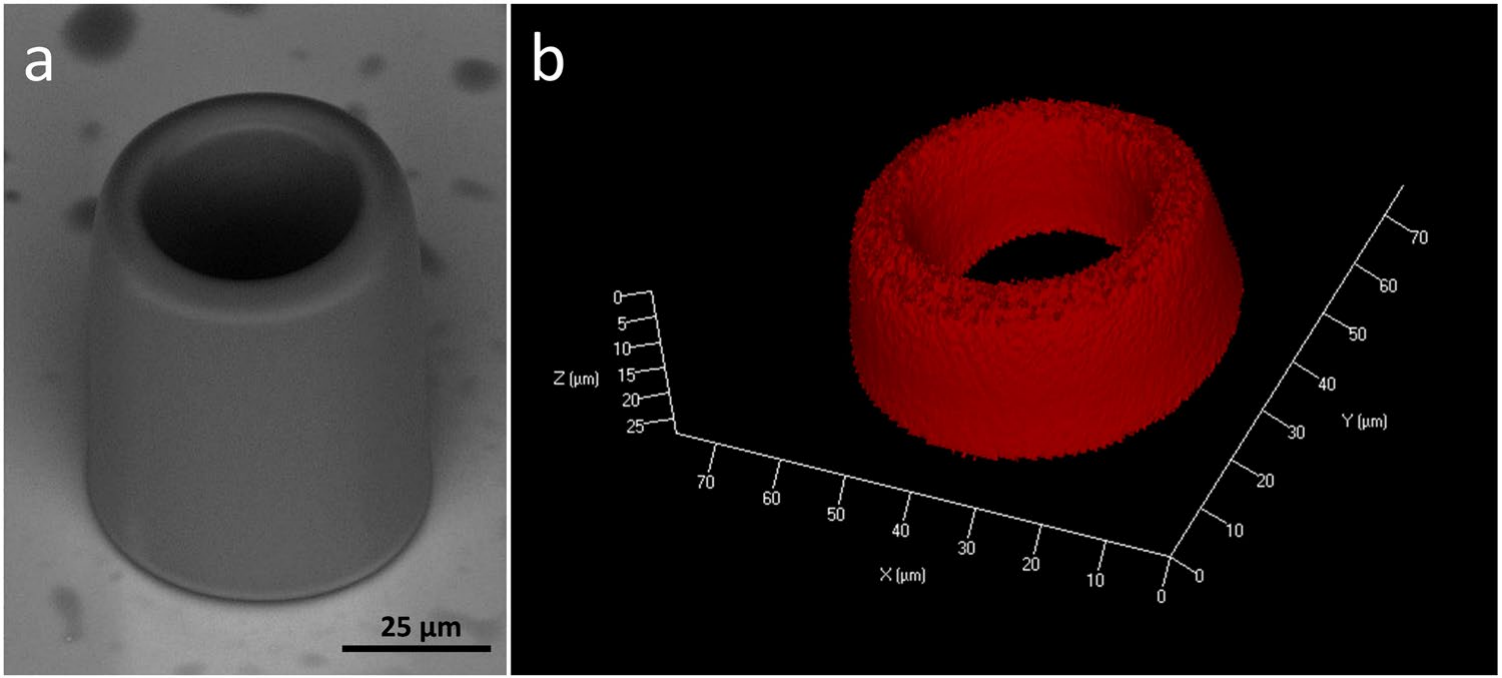
(**a**) Scanning electron and (**b**) 3D reconstructed confocal fluorescence micrographs of a Rhodamine B doped microcavity produced by two-photon polymerization.

Figure 1a reports a typical microcavity fabricated by employing 0.1 nJ pulses from a Ti:sapphire oscillator with 20 µm/s of laser scan speed. The hollow microcylinders exhibit an outer diameter of 50 µm, 6.5 µm of sidewall thickness and approximately 80 µm of height. As can be inferred from the scanning electron micrograph, they feature good structural integrity and smooth sidewall surface. The host material is a crosslinked acrylate polymer similar to the one reported previously^22^. Contrary to previous approaches^8, 23^, Rhodamine B is easily integrated into the polymer host material (concentration of 5.0 µmol/g of resin). Illustrated in Fig. 1b, the reconstructed confocal fluorescence micrograph confirms the dye is uniformly distributed throughout the top part of microcavity, which contributes mostly to lasing due to the exponential decay of excitation along the microstructure. Although there is a good match between the cross-sectional dimensions of both the scanning electron and the confocal micrograph, their height is clearly different. As fluorescence passes through the microcavity, it is re-absorbed by the dye molecules present in the close-to-top layers until it hits the detector. Consequently, for an integration time set to prevent signal saturation from the close-to-top layers, fluorescence from shorter layers is barely detected.

**Figure 2:**
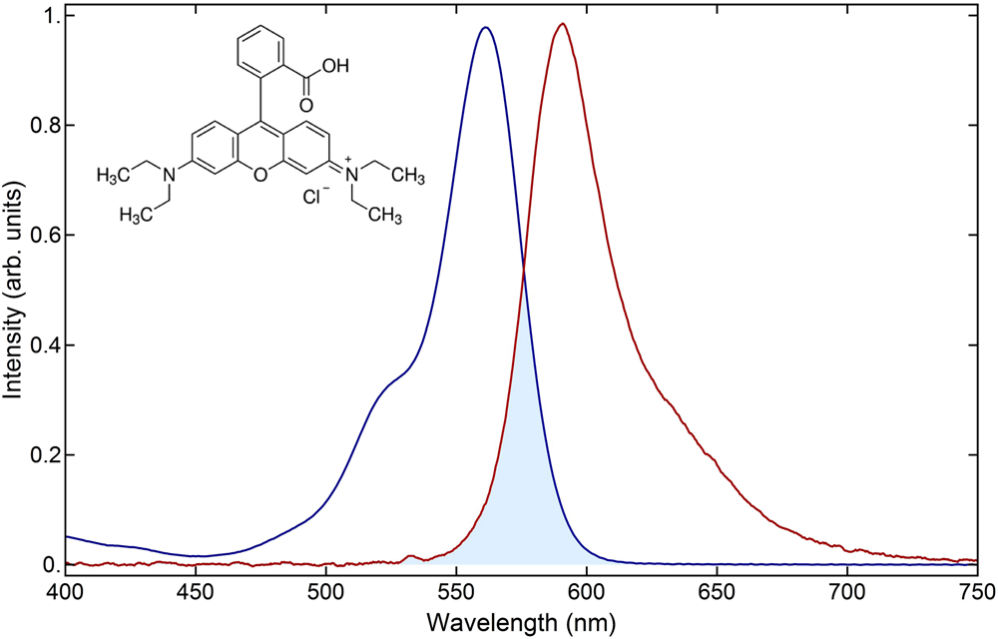
Absorption and fluorescence spectrum of an acrylate polymer sample (macroscopic) doped with Rhodamine B in a concentration of 5.0 µmol/g of resin. Highlighted in blue is the overlap between both spectra. The inset shows the chemical structure of Rhodamine B.

Depicted in Fig. 2 are the absorption/emission spectra of Rhodamine B dissolved in the acrylate polymer. They were compared to the spectra of a solution of RhB dissolved in ethanol at the same dye concentration as that used to prepare the doped photoresist. The polymeric matrix does not affect significantly the optical properties of the dye. The interaction with the acrylic polymer causes a 10 nm red shift of the dye absorption spectrum, though the emission spectrum remains unchanged.

**Figure 3:**
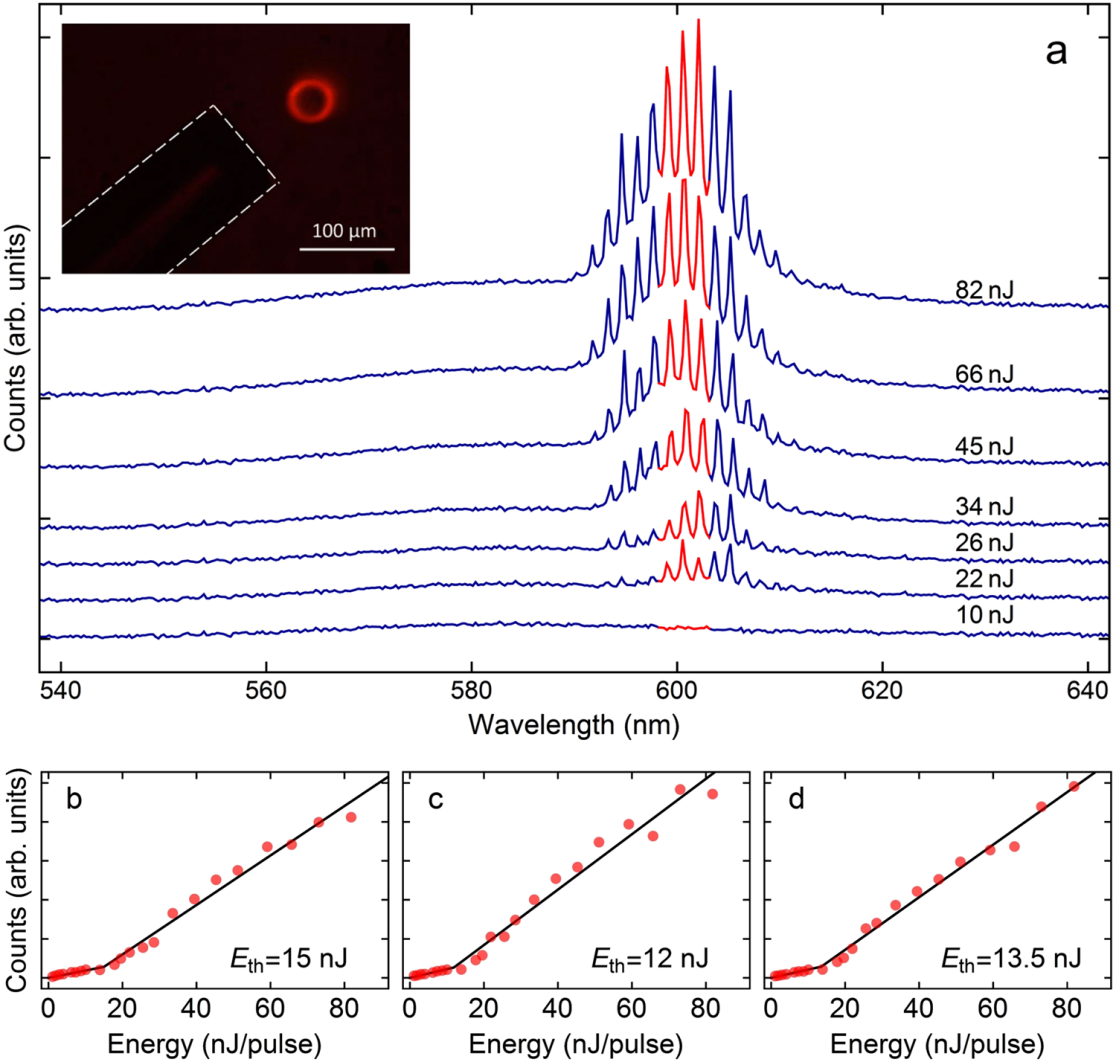
(**a**) Emission spectra of a Rhodamine B doped microcavity for the excitation energy levels indicated in the right hand side of the graph. To make the visualization easier, an offset was applied to the y-axis of each curve. The first curve represents the spectrum below lasing threshold. The inset shows an image of the microlaser being excited (82 nJ of input energy). Its emission is collected through the optical fiber (dashed outline to highlight). (**b**,**c** and **d**) Represents the growth of the peaks tagged in red in (**a**) as a function of excitation energy (threshold energies are shown in each corresponding graph).

The microcavities were excited from above with a 100-ps pulsed laser operating at 532 nm. Pulsed excitation was employed to prevent population transfer to the triplet lowest state, which would substantially drop the quantum yield of fluorescence of the dye^4^. Figure 3 shows emission curves acquired for different excitation energy. The spectra feature a set of peaks modulated by a Lorentzian-shape envelope with maximum at 600 nm and 10 nm of full width at half maximum (FWHM). Despite the broad emission spectrum of Rhodamine B, the peaks show up only within a spectral window from 590 to 620 nm. On the shorter wavelength side, the emission and absorption processes compete, thus hampering lasing effects at this spectral region.

The spectral spacing between consecutive modes within the FWHM of RhB emission spectra was compared to the microcavity free spectral range $FSR=\frac{{\lambda }^{2}}{2\,\pi \,r\,n\,(\lambda )}$, in which *λ* denotes the wavelength, *r* is the microcavity radius, and *n*(*λ*) is the effective refractive index at *λ* (n = 1.517 at 600 nm). The average FSR taken over all emission curves (FSR = 1.32 nm at 600 nm) matched the theoretical values within the instrumental error (0.25 nm of spectrometer resolution), thereby attributing lasing to the whispering gallery modes.

The loaded Q-factor, inferred from the peak linewidth, is 1.5 × 10^3^. However, as the peaks are not properly resolved by the spectrometer, we believe the Q-factor to be underestimated. In a previous study, we measured the loaded Q-factor of the undoped microcavities as 1 × 10^5^ at 1550 nm^22^. Their intrinsic Q-factor, limited mostly by absorptive and scattering losses, was found to be over 10^6^. One may expect the intrinsic Q-factor of the doped microcavities to not change significantly for visible wavelengths out of RhB absorption spectrum. At 600 nm, the attenuation coefficient may drop an order of magnitude (due to the lower absorption of the acrylate polymer at the visible spectrum), which scales up the absorption-limited Q-factor (*Q*_*abs*_). On the other hand, as the ratio $\lambda /\sigma$($\sigma \,\equiv$ surface roughness) grows for shorter wavelengths, the surface-scattering-limited Q-factor falls off^24^, thus compensating the rise of *Q*_*abs*_.

The bilinear behavior of the integrated intensity collected from the dye microlaser was obtained by plotting the peaks present in the emission spectra as a function of excitation energy (Fig. 3b–d). As lasing threshold depends on the wavelength, it varies slightly for different peaks. The lowest lasing threshold, measured at 12 nJ of pulse energy, occurred to the peak at the maximum of emission spectra. Such low threshold is comparable to what have been achieved for polymer microlasers fabricated by other techniques^9, 14, 25^ and is a direct result of the giant power density achieved within whispering gallery mode microcavities, combined with the high fluorescence quantum yield of Rhodamine B^26, 27^.

**Figure 4:**
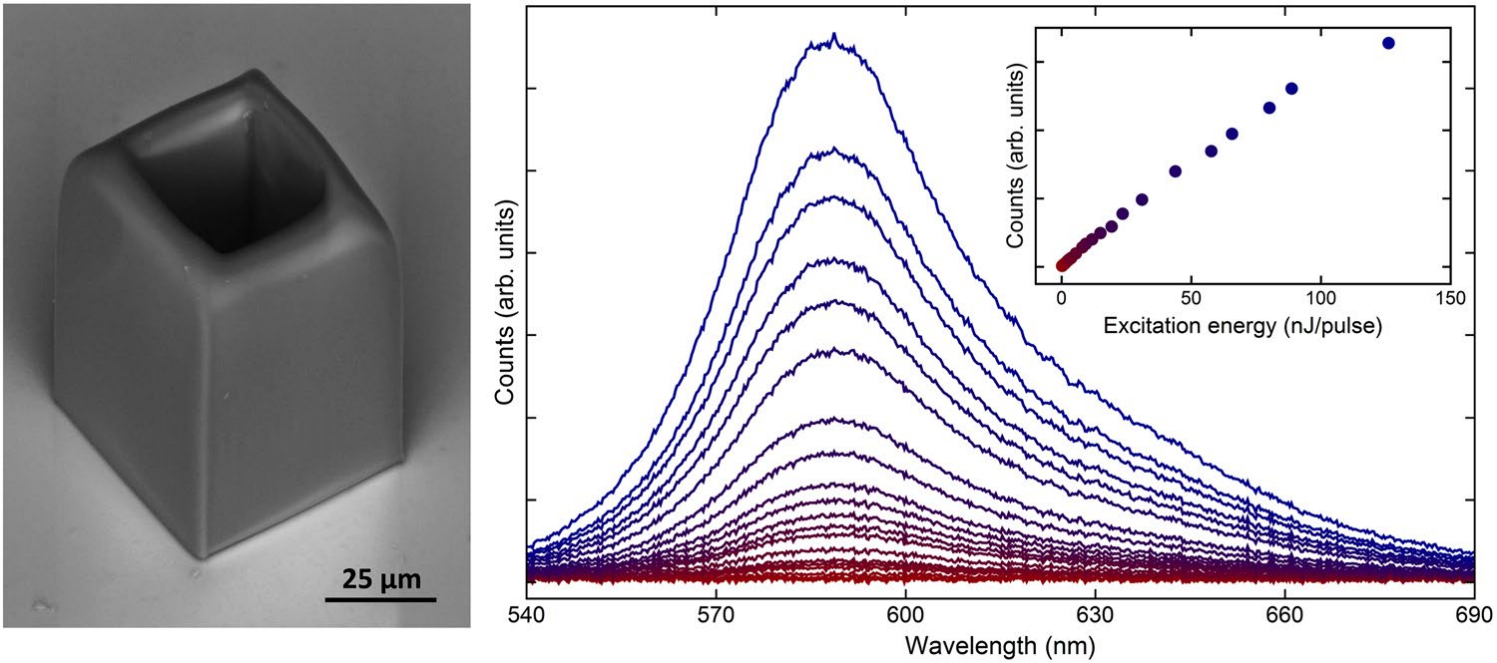
(**a**) Scanning electron of a hollow microcube doped with Rhodamine B (5.0 µmol/g of resin). (**b**) Emission spectra of the dye microcube depicted in (**a**) taken for several excitation energy levels. Indicated in the inset is the growth of the integrated emission intensity of the dye microcube as a function of excitation energy. The colour scale identifies the emission curves.

In principle, the crosslinked polymeric matrix may act as a scattering media, which combined with the presence of the dye, leads to random lasing effects^28^. To rule out such possibility, the emission spectra of RhB doped cubic hollow microstructures, which do not support WGMs, was compared with that of the microcylinders. The microcubes were fabricated by the same setup described herein and exhibit the same composition and volume of the microcylinders. As can be seen in Fig. 4, for the same pump energy levels as that used to excite the microcylinders, the spectra do not exhibit either peaks or a kink in the output versus input energy plot, confirming that random effects do not play a role in lasing threshold.

## Conclusion

In summary, laser action in dye-doped polymeric microcavities fabricated via femtosecond laser induced two-photon polymerization was demonstrated. Rhodamine B is easily integrated into the polymeric host matrix, which does not affect substantially the optical properties of the dye. As a result of their great surface optical quality and the low absorption of the acrylate polymer around 600 nm, the microlasers exhibited lasing threshold at a pump energy as low as 12 nJ for free space pulsed excitation at 532 nm. The calculated free spectral range is in good agreement with its measured counterpart, revealing that lasing occurs in whispering gallery modes. Besides, no lasing was observed in doped microcubes fabricated with the same concentration as that of the microcylinders, thus ruling out the influence of random lasing effects on the measured threshold. This work therefore represents a step forward in the field of soft photonics and demonstrates the potentiality of two-photon polymerization in fabricating high performance optical microdevices.

## Methods

Three dimensional microfabrication is performed using a negative-tone photoresist composed of two acrylate monomers and a photoinitiator. The monomers ethoxylated(6)trimethyl-lolpropanetriacrylate (SR499 - Sartomer^®^) and dipentaerythritol pentaacrylate (SR399 - Sartomer^®^) are mixed in a proportion of 10/90 wt%. The photoinitiator is the 2,4,6-trimethylbenzoylphenyl phosphinate, an acylphosphine oxide photoinitiator commercially known as Lucirin TPO-L (in excess of 3 wt%, Igarcure^®^). RhB (Sigma-Aldrich^®^) is first dissolved in ethanol and added to the polymeric resin in a concentration of 5.0 µmol/g of resin. The solution is mixed for 30 min and left to rest by 48 hours previously to fabrication. Once the solvent has evaporated, the photoresist is dropped onto a glass substrate and covered by a cover slip with a spacer. The sample is then placed on a translation stage that is mounted on an inverted microscope. 100-fs pulses of a mode-locked Ti:sapphire oscillator operating at 780 nm are focused into the volume of the polymeric resin using a NA 0.25 objective lens. The microstructures are created by controlling both a galvanometric-mirror system and the stage that supports the photopolymer with computer-aided software. For a detailed description of the microfabrication process, the reader is referred to ref. 22.

The apparatus assembled for analyzing the emission of both the microcylinders and microcubes uses as excitation source a frequency-doubled (532 nm) Q-switched/mode-locked Nd:YAG laser operating at 100 Hz, which delivers a sequence of 100-ps pulses modulated by the Q-switched envelope (pulse train). The excitation was carried out at room temperature. To assure single pulse operation, a Pockels cell and a polarizer are added to the system. The laser beam is focused with a 7.5 cm focal length lens, resulting in a beam waist with 50 µm of radius on the microcavity top surface. Microcavity emission is acquired with a conventional optical fiber connected to a spectrometer (*Ocean Optics HR4000* ^®^). The sample (microcavities stood on a glass substrate) rests on an inverted microscope coupled to a CCD camera that allows real time monitoring of the excitation process. A half-wave plate, combined with a polarizer, is used to adjust excitation laser power, allowing exciting the microcavities with different energy levels.

The microcavities were characterized by scanning electron (*Hitachi TM3000* ^®^) and z-stack confocal fluorescence microscopies (*Zeiss LSM 700* ^®^, 40x Objective lens, excitation at 445 nm, 1 µm of z-step). The absorbance of RhB dissolved in both ethanol and acrylic polymer and the refractive index of the acrylic polymer around 600 nm were measured with the help of a *Shimadszu UV-1800* ^®^ spectrometer and a *Carl Zeiss Jena Pulfrich Refractometer PR2* ^®^, respectively. Fluorescence spectra were collected on the same setup used to analyze microcavity emission. These measurements were performed in macroscopic samples of RhB-doped acrylic polymer with the same composition used to produce the microcavities.

### Data Availability

All data generated or analyzed during this study are included in this published article.

## References

[1] Schafer FP, Schmidt W, Marth K (1967). New dye lasers covering visible spectrum. Phys. Lett. A..

[2] Sorokin PP, Lankard JR, Hammond EC, Moruzzi VL (1967). Laser-pumped Stimulated Emission from Organic Dyes: Experimental Studies and Analytical Comparisons. IBM J. Res. Dev.

[3] Soffer BH, McFarland BB (1967). Continuously tunable narrow-band organic dye lasers. Appl. Phys. Lett..

[4] Schäfer, F. P. Dye Lasers **34**, 97 (Springer, 1990).

[5] Thiel E, Zander C, Drexhage KH (1986). Continuous wave dye-laser pumped by a hene laser. Opt. Commun..

[6] He LN, Ozdemir SK, Yang L (2013). Whispering gallery microcavity lasers. Laser Photon. Rev.

[7] Samuel IDW, Turnbull GA (2004). Polymer lasers: recent advances. Mater. Today.

[8] Tulek A, Akbulut D, Bayindir M (2009). Ultralow threshold laser action from toroidal polymer microcavity. Appl. Phys. Lett..

[9] Grossmann T (2010). Low-threshold conical microcavity dye lasers. Appl. Phys. Lett..

[10] Grossmann T (2011). Direct laser writing for active and passive high-Q polymer microdisks on silicon. Opt. Express.

[11] Parsanasab GM, Moshkani M, Gharavi A (2015). Femtosecond laser direct writing of single mode polymer micro ring laser with high stability and low pumping threshold. Opt. Express.

[12] Das AJ, Lafargue C, Lebental M, Zyss J, Narayan KS (2011). Three-dimensional microlasers based on polymer fibers fabricated by electrospinning. Appl. Phys. Lett..

[13] Frolov SV, Shkunov M, Vardeny ZV, Yoshino K (1997). Ring microlasers from conducting polymers. Phys. Rev. B.

[14] Zhang C (2015). Organic printed photonics: From microring lasers to integrated circuits. Sci. Adv.

[15] Guo R (2006). Micro lens fabrication by means of femtosecond two photon photopolymerization. Opt. Express.

[16] Seet KK, Mizeikis V, Matsuo S, Juodkazis S, Misawa H (2005). Three-dimensional spiral-architecture photonic crystals obtained by direct laser writing. Adv. Mater..

[17] Liu Z-P (2010). Direct laser writing of whispering gallery microcavities by two-photon polymerization. Appl. Phys. Lett.

[18] LaFratta CN, Fourkas JT, Baldacchini T, Farrer RA (2007). Multiphoton fabrication. Angew. Chem. Int. Edit.

[19] Mendonca CR (2009). Three-dimensional fabrication of optically active microstructures containing an electroluminescent polymer. Appl. Phys. Lett..

[20] Correa DS (2009). Two-Photon Polymerization for Fabricating Structures Containing the Biopolymer Chitosan. J. Nanosci. Nanotechnol.

[21] Sun HB, Tanaka T, Takada K, Kawata S (2001). Two-photon photopolymerization and diagnosis of three-dimensional microstructures containing fluorescent dyes. Appl. Phys. Lett..

[22] Tomazio NB (2017). Femtosecond laser fabrication of high-Q whispering gallery mode microresonators via two-photon polymerization. J. Polym. Sci. Part B: Polym. Phys.

[23] Min B (2006). Ultralow threshold on-chip microcavity nanocrystal quantum dot lasers. Appl. Phys. Lett..

[24] Gorodetsky ML, Savchenkov AA, Ilchenko VS (1996). Ultimate Q of optical microsphere resonators. Opt. Lett.

[25] Grossmann T (2011). Strongly confined, low-threshold laser modes in organic semiconductor microgoblets. Opt. Express.

[26] Kubin RF, Fletcher AN (1982). Fluorescence quantum yields of some rhodamine dyes. J. Lumin..

[27] Snare MJ, Treloar FE, Ghiggino KP, Thistlethwaite PJ (1982). The photophysics of rhodamine B. J. Photochem..

[28] Wiersma DS (2008). The physics and applications of random lasers. Nat. Phys.

